# Pharmacogenomics Guided Prescription Changes Improved Medication Effectiveness in Patients With Mental Health-Related Disability: A Retrospective Cohort Analyses

**DOI:** 10.3389/fgene.2021.644694

**Published:** 2021-08-03

**Authors:** Sanjida Ahmed, Ramzan Tahir, Umbreen Akhtar, Mark Faiz

**Affiliations:** ^1^Personalized Prescribing Inc., Toronto, ON, Canada; ^2^Apotex Pharmaceuticals, Toronto, ON, Canada; ^3^Canadian College of Healthcare and Pharmaceutics, Toronto, ON, Canada

**Keywords:** pharmacogenomics, genetic mutation, morbidity, disability, mental health

## Abstract

Mental health problems are the leading cause of disability in Canadian workers. Medication ineffectiveness is hypothesized to increase the time to return-to-work in these workers. We assessed whether prescription changes based on pharmacogenomics profiling (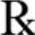 Report^®^) improved medication effectiveness in patients on mental health-related disability. In this retrospective cohort analyses, we assessed the impact of pharmacogenomic profiling on patient outcomes in 84 Canadian workers who were on a mental health-related disability between May 2018 and May 2019. All patients completed an informed consent form and a standard questionnaire including medical history, medications, disease symptoms, and medication side effects. Licensed pharmacists made recommendations for prescription changes in 83 patients. The main study outcome was medication effectiveness defined on a scale of 0 to 10 (0 being most effective and 10 being most ineffective) based on reported mood toward regular work tasks and medication side effects. We compared the medication effectiveness at baseline and at 3 months after the pharmacogenomics-based prescription changes. This retrospective cohort analyses included 46 patients who completed the follow-up questionnaires. Of them, 54% (*n* = 25) were females, 67% (n = 31) were Caucasians, and the mean age was 38 years (standard deviation [SD] = 11). The average baseline effectiveness score was 8.39 (SD =1.22). Following the prescription changes, the medication effectiveness scores significantly improved to an average of 2.30 (SD = 1.01) at 3 months of follow-up (effect size *r* = 0.62, *p* = <0.001). Pharmacogenomics could help in improving treatment outcomes in patients on mental health-related disability.

## Introduction

Mental health problems are the leading cause of disability among the Canadian workers (Lim et al., 2008; Mental Health Commission of Canada, 2012; Institute for Health Metrics Evaluation, 2018). The Mental Health Commission of Canada estimates that about one in three workplace disability claims is related to mental illnesses amounting to 70% of the total disability costs (Mood Disorder Society of Canada, 2009; Towers Watson, 2011; Risk Analytica Mental Health Commission of Canada, 2013; RBC Life Insurance Company, 2016). Only 50–60% of patients suffering from mental health conditions respond to prescribed medications the first time (Gardner et al., 2014; Alboni et al., 2016). Most of these patients experience side effects from psychotropic medications (Ghoshal, 2019).

Pharmacogenomics plays a major role in medication effectiveness in patients with mental health conditions (Singh, 2015). According to the Thousand Genome Project, at least 40% of the general population have genetic variations that predispose them to treatment ineffectiveness and toxicity if they are prescribed mental health-related medications (1000 Genomes Project, 2013). A patient based on their pharmacogenomic profile can be on the spectrum of “poor metabolizer (PM),” “intermediate metabolizer (IM),” “extensive (normal) metabolizer,” and “ultra-rapid metabolizer (UM)” for a medication (English et al., 2012). For instance, a patient who is an ultra-rapid metabolizer would require increased dosage for that medication. Conversely, a patient who is a poor metabolizer may have more side effects on that medication. These phenotypes are classified as pharmacokinetic effects and are predicted by variations in CYP2D6 and CYP2C19 genes that affect the metabolism of most psychotropic medications (Pouget et al., 2014). Similarly, genetic variations also moderate the pharmacodynamics of medications, i.e., how the drug transport, target receptors, and cell signaling augment or diminish the effects of a medication.

Recent work shows the value of genomic profiling in patients with mental health-related prescriptions. For example, a Mayo Clinic study reported that prescription changes after a commercial pharmacogenetic test (GeneSight^®^) improved treatment outcomes in about 114 patients of major depressive disorder (Hall-Flavin et al., 2013; Perez et al., 2017; Tanner et al., 2018). Another correlation study of 96 patients suffering from mood disorders showed that those who had poor genetic profiles for drug metabolism had 69% higher healthcare use, three-fold more work absences, and four-fold more increases in disability claims (Winner et al., 2013). Literature is relatively silent about the potential impact of pharmacogenomics-based prescription changes on treatment outcomes in workers on mental health-related disability (Fischer, 2005; Kim-Lian et al., 2008; Smetanin, 2011). Psychiatrists and other healthcare providers are yet to utilize pharmacogenomics in clinical practices, though there are 13 pharmacogenetic tests available in Canada relevant to psychiatry (Maruf et al., 2020). Recent systemic reviews, meta-analysis and randomized control trials indicate the probability of positive impact on symptom remission for individuals suffering from moderate to severe depression, however, no real-world database studies are available on effectiveness of these tests in practice (Li and Loshak, 2020; Tanner et al., 2020; Papastergiou et al., 2021). Such investigation could help employers to consider whether such interventions could improve return-to-work related indicators in their workers on mental health-related disability.

We have developed a specific pharmacogenomic test, the 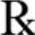 Report^®^, for recommending prescription changes in patients who have a mental health-related disability. The test takes into account 54 genes. The aim of this present study was to assess whether the prescription changes following the 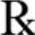 Report^®^ improved medication effectiveness in workers on mental health-related disability.

## Methods

### Study Design and Setting

We conducted a retrospective cohort analyses of patients who were residing in Canada and were on mental health-related disability. The patients had prescription changes following pharmacogenomic profile using the 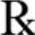 Report^®^. The main outcome measure was treatment effectiveness assessed by a licensed pharmacist at baseline and at 3 months. All patients signed informed consent forms for their data to be used for evaluation. As per the article 2.5 of the Tri-Council Policy Statement 2 of the Canadian human research ethics guidelines, the present analyses qualified as a quality assurance study. The protocol was reviewed by the Research Ethics Board of the Sunnybrook Health Sciences Centre (Toronto, ON) with reference # PIN 3336 (Approved Nov 30, 2020). The Sunnybrook REB has determined that an Informed Consent Form (ICF) is not required for this study.

### Participants

For this study, we included patients who were employed and had a mental health-related disability and they were referred by their disability managers from May 2018 to May 2019. The case managers conducted an independent medical evaluation for each worker and referred patients for 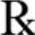 Report^®^ when they suspected that the prescribed treatment change might be warranted.

### Procedures

All patients were contacted by a licensed pharmacist to discuss their medication history. The pharmacist used a pre-defined questionnaire evaluating treatment effectiveness including side effects and mood toward regular work tasks. A DNA saliva sample kit was shipped to each patient for sample collection. Patients were directed to collect their saliva samples and ship them to test facility. The laboratory processed the saliva samples, extracted DNA, amplified DNA using polymerase chain reaction (PCR) and reported the genetic variations using Agena Bioscience Mass Array system. The results were interpreted using the proprietary software containing an algorithm of 104 key genetic variations from 54 genes (see Table 1 for details). Based on results, the 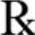 Report^®^ was generated recommending prescription changes. The online access information to view the test report was forwarded to patients. Copies of the pharmacist-report including recommendations for prescription changes were sent to patient's physician and the disability case manager. The report did not include any genetic information. After 3 months, the same pharmacist contacted the patients to evaluate their treatment effectiveness.

**Table 1:** List of genes and variants tested under 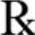 Report^®^ test panel.

**No**.	**Gene**	**SNPs**
1.	CYP2C19	rs12248560, rs28399504, rs41291556, rs4244285, rs4986893, rs56337013, rs72552267, rs72558186
2.	CYP2C9	rs9332239, rs9332131, rs7900194, rs72558190, rs72558189, rs28371686, rs1799853, rs1057910, rs28371685, rs56165452
3.	CYP2D6	rs59421388, rs5030867, rs5030865, rs5030656, rs5030655, rs3892097, rs35742686, rs28371725, rs5030862, rs16947, rs28371735, rs1065852, rs5030863, rs28371706, rs72549357
4.	CYP2B6	rs2279343, rs3211371, rs3745274, rs8192709, rs28399499,
5.	F5	rs6025
6.	SLCO1B1	rs4149056
7.	VKORC1	rs9923231, rs9934438
8.		rs2952768
9.	CYP1A2	rs2069514, rs762551
10.	CYP3A4	rs35599367
11.	CYP3A5	rs776746
12.	APOE	rs7412
13.	ABCB1	rs1045642, rs2032583
14.	ADRA2A	rs1800544
15.	ADRB1	rs1801253, rs1801252
16.	ADRB2	rs1042713
17.	BDNF	rs6265
18.	CACNA1C	rs1006737
19.	CNR1	rs806368, rs1049353
20.	CHRNB2	rs2072661
21.	COMT	rs13306278, rs165599, rs6269, rs4680
22.	COQ2	rs4693075
23.	DRD1	rs4532
24.	DRD2	rs1799732, rs1799978, rs1800497
25.	DRD3	rs6280
26.	FAAH	rs324420
27.	FKBP5	rs4713916
28.	GNB3	rs5443
29.	GRIA1	rs1994862
30.	GRIK4	rs1954787
31.	HLA-B	rs2395029, rs2844682
32.	HSPG2	rs2445142
33.	HTR1A	rs6295, rs10042486, rs1364043
34.	HTR2A	rs6313, rs7997012
35.	HTR2C	rs3813929, rs1414334
36.	MC4R	rs17782313
37.	MTHFR	rs1801133, rs1801131
38.	NEDD4L	rs4149601
39.	OPRD1	rs529520
40.	OPRM1	rs2952768, rs1799971
41.	PRKCA	rs16960228
42.	POLG	rs3087374
43.	RGS4	rs951439
44.	SACM1L	rs2742417
45.	SLC6A4	l or s, rs25531
46.	SLC6A2	rs2242446
47.	SCN1A	rs3812718
48.	SCN2A	rs17183814
49.	TPH1	rs1800532
50.	TPH2	rs1487278
51.	UGT1A4	rs2011425
52.	UGT2B15	rs1902023, rs2952768
53.	YEATS4	rs7297610
54.	ZNF804A	rs1344706

### Measures

The main outcome measure was the treatment effectiveness score ranging from 0 (Good) to 10 (Worst). The score was divided in two parts. In the first part, the patient was asked about mood toward regular life or work-related task on a scale of 1–5. The patient ranked the score as “1” for “I feel good,” 2–3 for “I force myself to do work” and 4–5 for “I am unable to work.” In the second part, the patient was asked to report any side effects of the medication. A maximum score of 5 was reported for side effects. We added both scores to compute treatment effectiveness.

We computed the genetic mutation scores based on degree of genetic variations identified for CYP2D6 and CYP2C19. If the genetic variation indicated an extreme profile, i.e., ultra-rapid or poor metabolizer, then a maximum score of 5 was assigned. If the variation indicated a profile of intermediate metabolizer, a score of 2 was assigned. Normal metabolizers for any of the gene were scored as 0. An individual having extreme variations in CYP2D6 and CYP2C19 (ultra-rapid or poor), a score of 10 was assigned. We also considered genetic variations in the brain receptor genes, (i.e., HTR1A, HTR1B, HTR2A, DRD2, ADRA2A etc.) including pharmacodynamic genes (i.e., BDNF, COMT, TPH2) and transporter genes (i.e., ABCB1) etc. In this case, for each variation, in receptor, transporter and other genes, we added a score of one up to a maximum of five. Hence, the genetic mutation score ranged from 0 to 15. Of note, the differences in scores were based on the effect sizes of genetic variations on drug pharmacokinetics and pharmacodynamic.

Other patient factors considered in the analyses were patient's age, sex, and ethnicity.

### Statistical Analysis

Descriptive statistics (mean and standard deviation for continuous variables and frequencies for categorical variables) were computed for patients. Due to the nature of scoring data, non-parametric statistical tests were used for the analyses. Wilcoxon-sign rank test was used for comparisons between baseline and follow-up for the treatment effectiveness. The strength of relationship between the genetic mutation score and treatment effectiveness score was computed by Spearman's rank correlation coefficient. Effect size (r) for Wilcoxon test was considered large if it is >0.05. Lastly, we plotted the receiver operating characteristic (ROC) curve to assess the accuracy of the genetic mutation score in predicting treatment effectiveness.

## Results

### Demographic Characteristics

**Figure 1:**
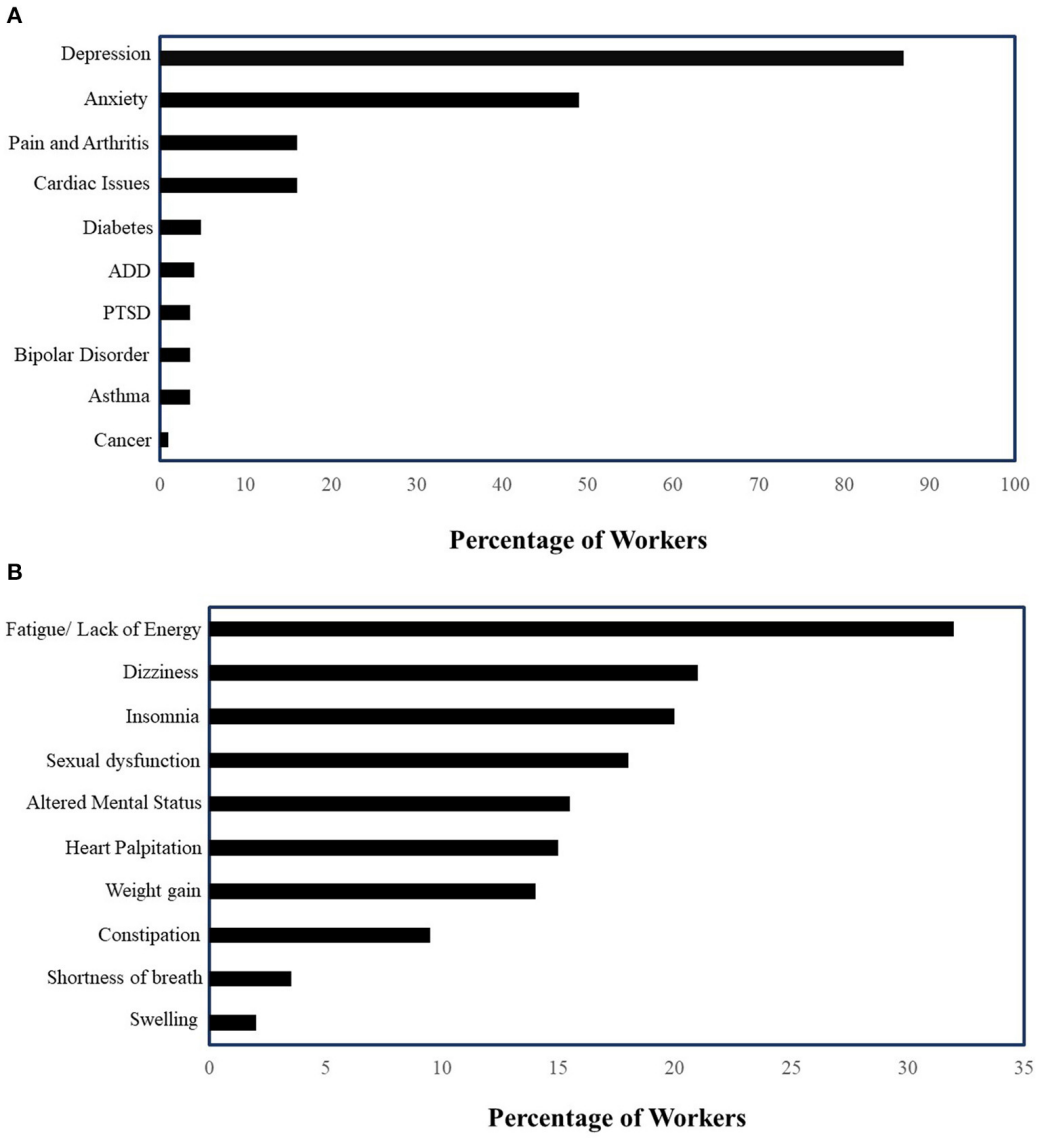
**(A)** Prevalence of mental health conditions in patients on mental health-related disability (*n* = 84) (PTSD, Post-traumatic Stress Disorder; ADD, Attention Deficit Disorder). **(B)** Medication-related side effects in patients on mental health-related disability (*n* = 84).

Eighty-four patients underwent a baseline assessment and genetic tests. The cohort analyses included only 46 patients who completed the follow-up assessment. The mean age of patients was 35 years (standard deviation = 11) in the complete cohort and 37 years (SD = 11) in the cohort with follow-up assessment (Table 2). Women accounted for 52% of patients (*n* = 37) in the complete cohort and 39% (*n* = 18) in the follow-up cohort (Table 2). About 69% (*n* = 48) patients were Caucasians, 6% (*n* = 5) were Asians in the complete cohort, proportions that were similar in the follow-up cohort (Table 2). Almost all patients (95%) were on one or more medications. The most common type of condition recorded was depression and anxiety (Figure 1A), and common medication-related side effects included fatigue (32%), dizziness (17%), insomnia (17%), sexual dysfunction (17%), and weight gain (12%) (Figure 1B).

**Table 2:** Characteristics of patients on mental health-related disability.

	**All patients (*n* = 84)**	**Patients with complete follow-up (*n* = 46)**
Sex		
Female, n (%)	45 (52.4)	18 (39.1)
Male, n (%)	38 (42.9)	25 (54.3)
Ethnicity		
Asian, n (%)	5 (6.0)	3 (6.5)
Caucasian, n (%)	58 (69.0)	31 (67.4)
Other, n (%)	3 (3.6)	3 (6.5)
Pharmacogenomic profile		
Both, n (%)	64 (76.2)	30 (65.2)
Pharmacodynamic, n (%)	2 (2.4)	1 (2.2)
Pharmacokinetic, n (%)	27 (20.2)	15 (32.6)
Age in years, Mean (SD)	35.5 (10.6)	37.1 (10.8)
Genetic mutation score, Mean (SD)	7.92 (2.20)	8.07 (2.06)

### Genetic Mutation Scores

**Figure 2:**
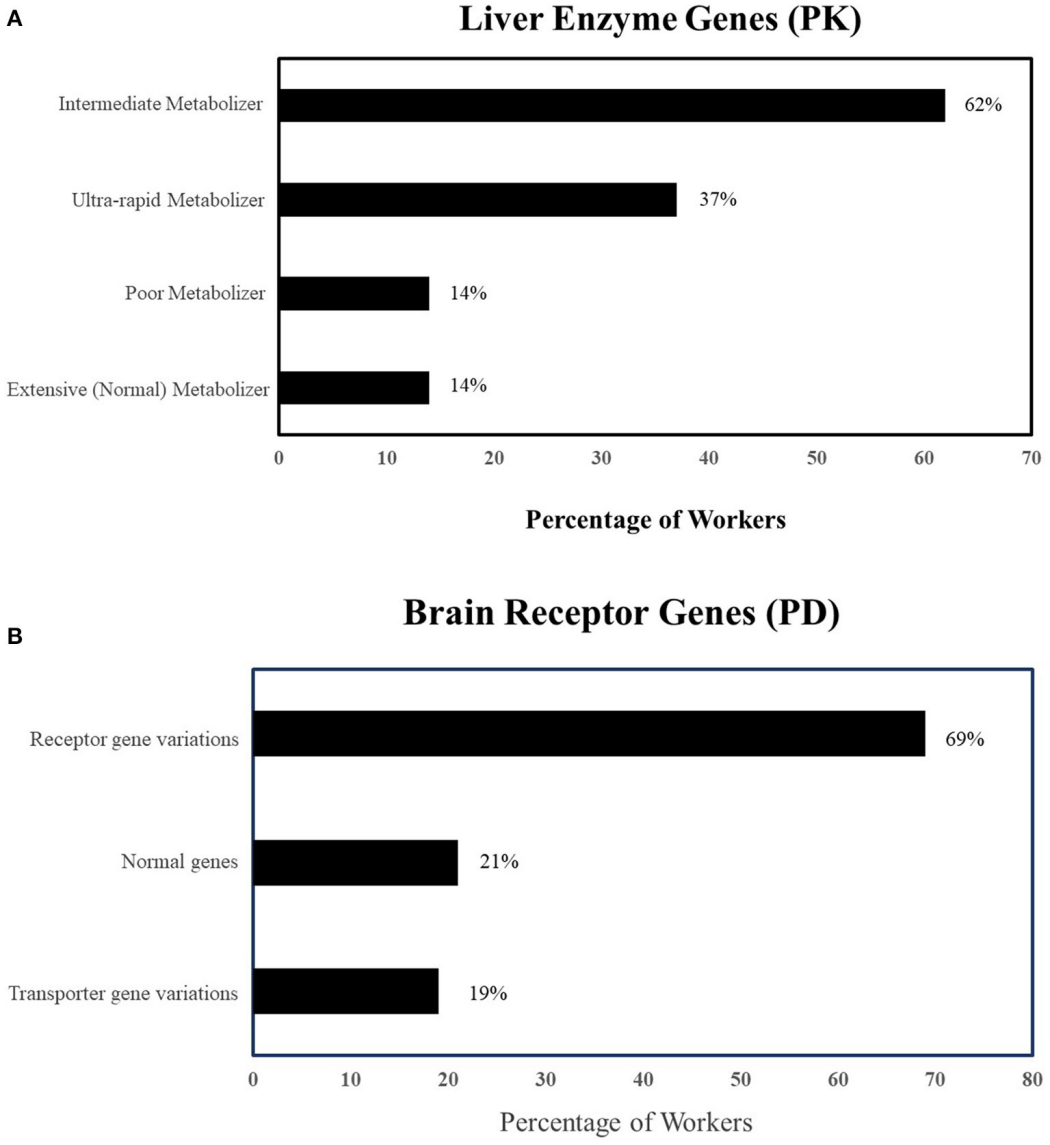
**(A)** Prevalence of pharmacokinetic genetic variations in 84 patients on mental health-related disability (PK, Pharmacokinetics; UM, Ultra-rapid metabolizer; IM, Intermediate metabolizer; PM, Poor metabolizer). **(B)** Prevalence of pharmacodynamic genetic variations in 84 patients on mental health-related disability. PD, Pharmacodynamics, Mutated Receptors: HTR2A, DRD2 etc.; Mutated Transporters: ABCBI.

We calculated genetic mutation scores for each patient based on the genetic mutation scores defined in the process. The results showed that all 84 patients had one or more genetic variations in their pharmacokinetic or pharmacodynamic genes. This implies they all had genetic mutation score of five or more than five. Of the patients who had CYP2C19 and CYP2D6 mutations (*n* = 71), 14% were poor metabolizers, 37% were ultra-rapid metabolizer, and 62% are intermediate metabolizers of one or both genes (Figure 2A). About 69% of patients had mutations for brain receptor genes (e.g., HTR2A, DRD2) and 19% had transporter gene-related mutations (e.g., ABCB1) (Figure 2B). About 66% of patients had mutations in both pharmacokinetic and pharmacodynamic genes.

### 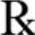 Report^®^ Test Utilization Outcome

**Figure 3:**
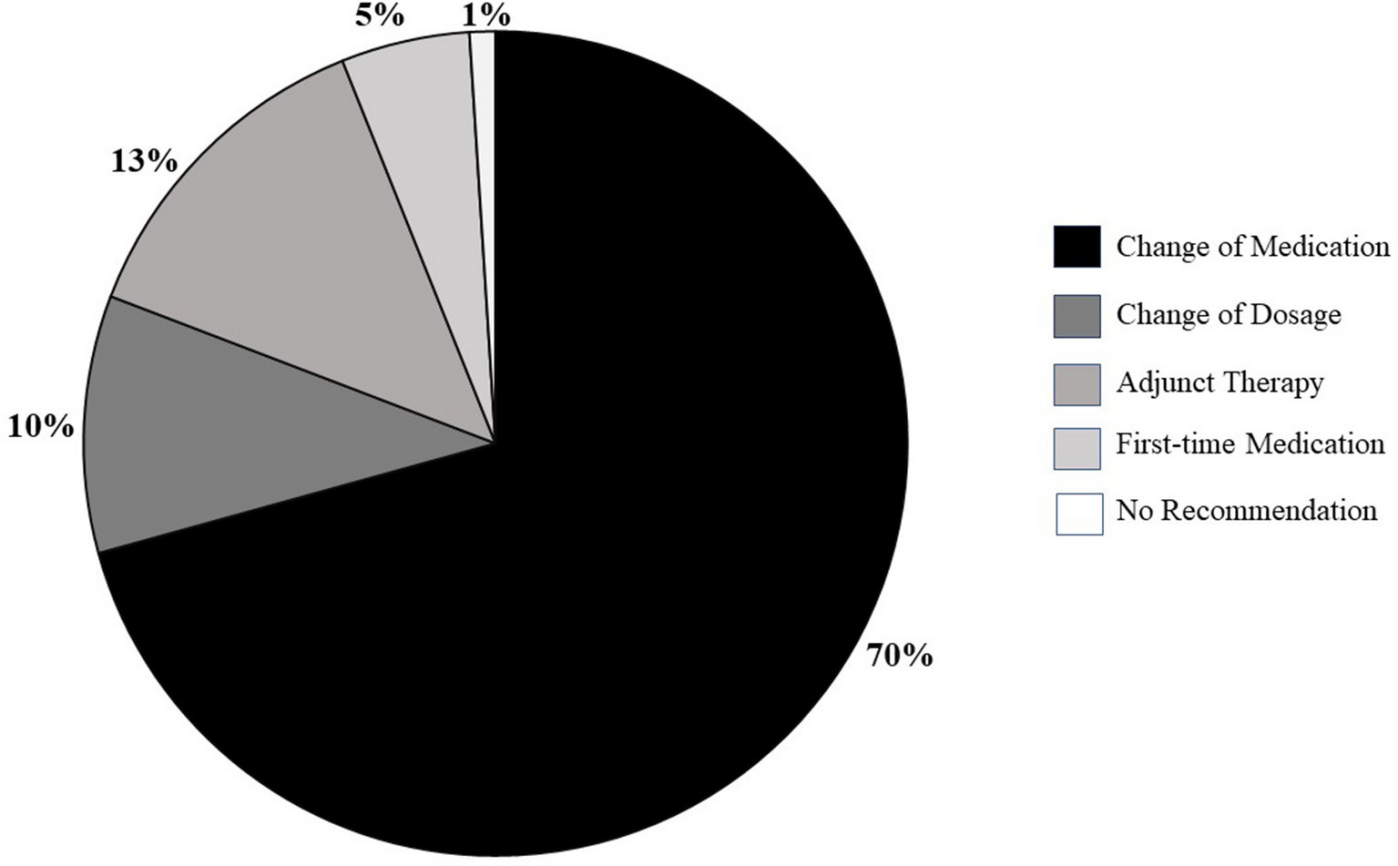
Types of prescription changes recommended in 46 patients on mental health-related disability.

Forty-six patients were followed up after they completed the 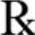 Report^®^ pharmacogenomic test. The prescription medication was changed in 70% of the patients based our recommendation. In other patients, dosage was modified in 10% (*n* = 8), adjunct medication was added in 13% (*n* = 11), and a new medication was prescribed in 5% (*n* = 4) who were not on any medications. Only one patient did not require any change (Figure 3).

### Treatment Effectiveness Score Changes

**Figure 4:**
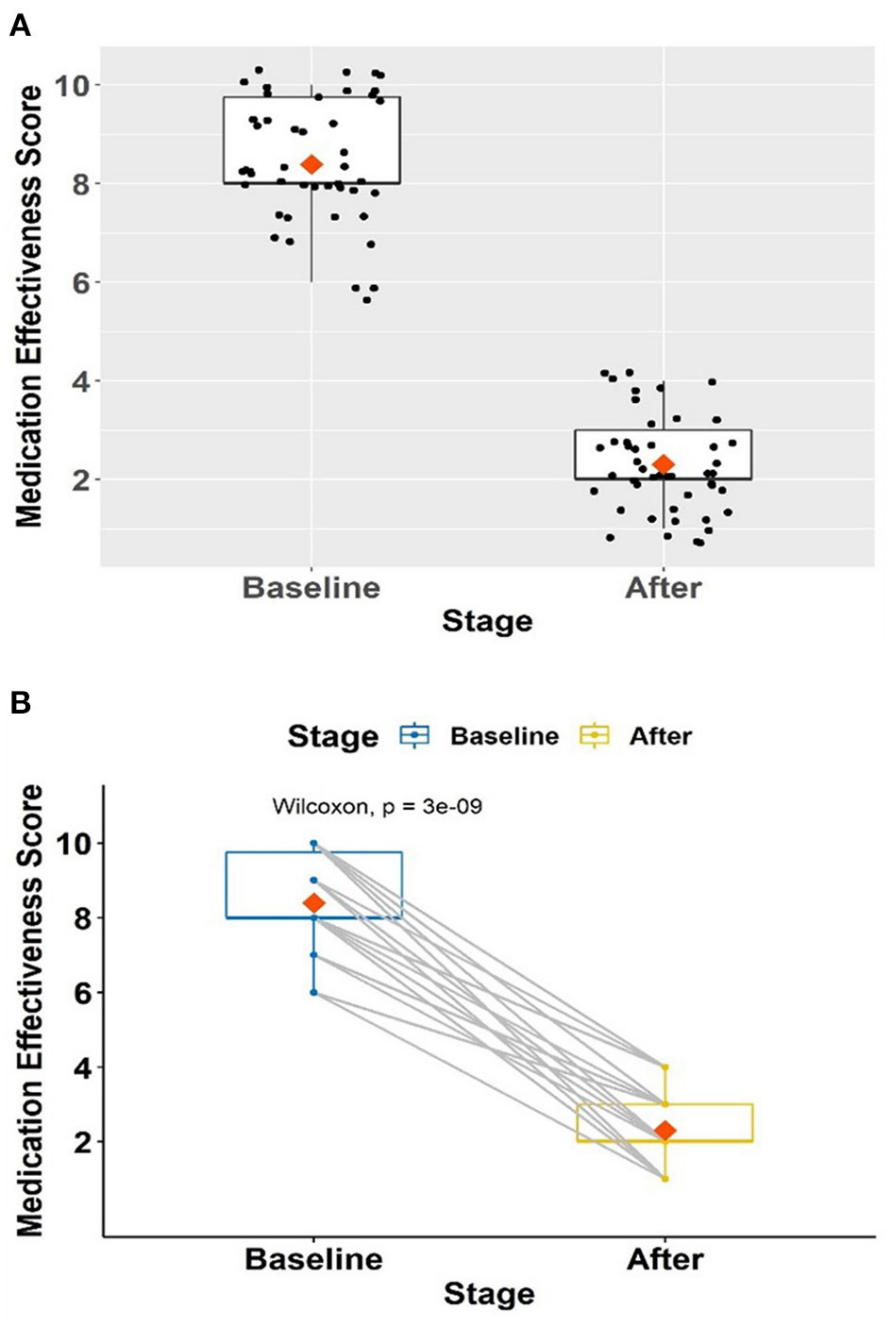
**(A,B)** Change in treatment effectiveness scores in 46 patients on mental health-related disability.

The average baseline effectiveness score was 8.39 (SD = 1.22) (Figures 4A,B). Following the prescription changes, the medication effectiveness scores significantly improved to an average of 2.30 (SD = 1.01) at 3 months of follow-up (effect size, *r* = 0.62, *p* = <0.001).

### Correlation Between Genetic Mutation and Baseline Treatment Effectiveness Score

**Figure 5:**
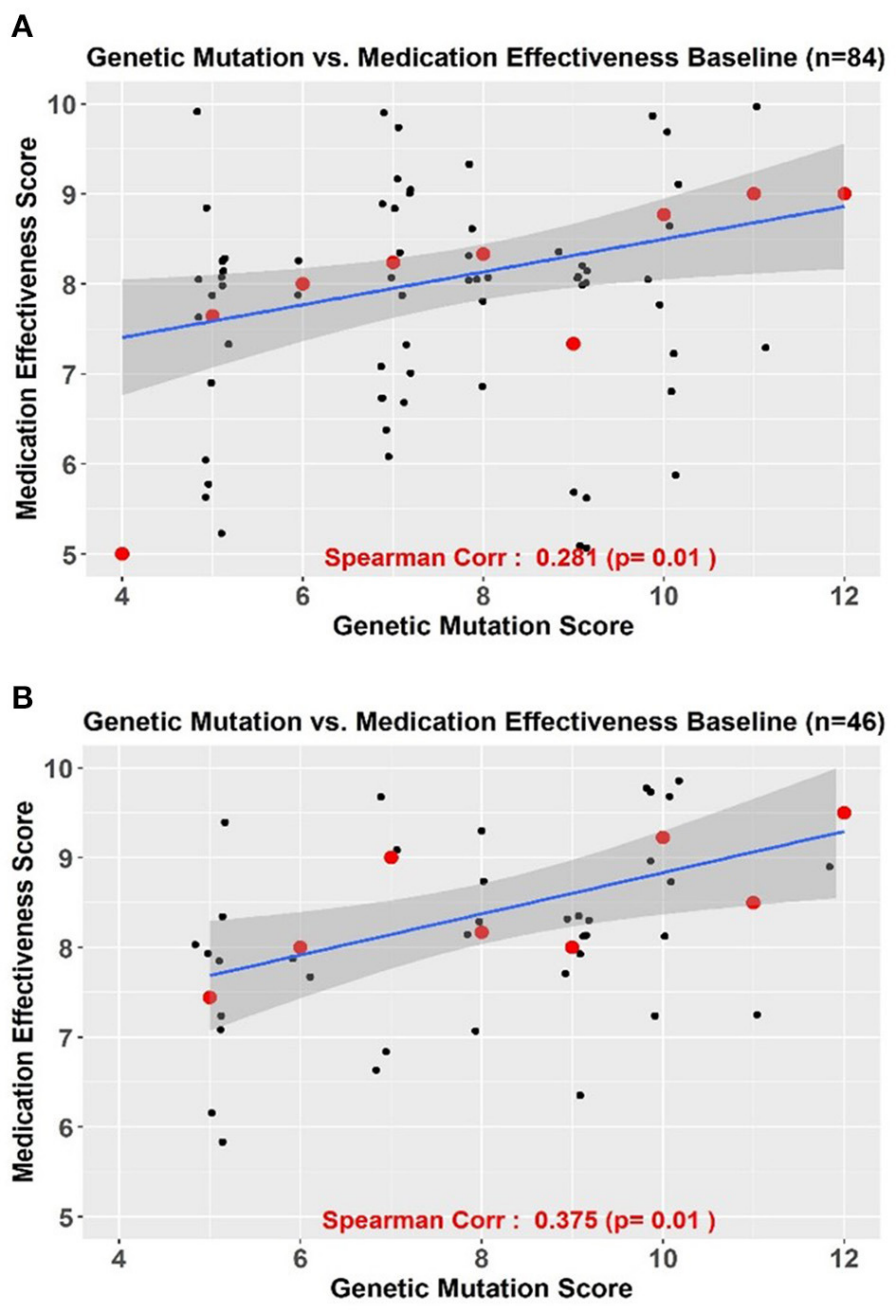
**(A,B)** Correlation between genetic mutation scores and treatment effectiveness scores in all patients **(A)** *n* = 84 and in those with complete follow-up **(B)** *n* = 46.

In the supplementary analyses, we found a significant correlation between genetic mutation and baseline treatment effectiveness scores for 84 patients (Spearman's correlation coefficient = 0.281, *p* = 0.01) (Figure 5A). Similar trends were seen in patients who completed follow-up (Spearman's Correlation coefficient = 0.375, *p* = 0.01) (Figure 5B). Using the bootstrapping method, a smooth roc curve was plotted with an AUC = 0.703 suggesting that 70% of the high treatment effectiveness score could be predicted by the genetic mutation score (data not shown).

## Discussion

This report has assessed treatment outcomes in workers with mental health-related disability following prescription changes resulting from pharmacogenomic profiling (the 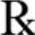 Report^®^). We show marked improvements in mood toward regular life and work tasks, and side effects in these patients. These findings could have significant implications for the workers and their employers in terms of earlier return-to-work and reduced disability costs.

Canada has one of the highest per capita usage rates of psychotropic medications globally (Fischer, 2005). Psychotropic medications are commonly prescribed in workers with mental health-related conditions. These psychotropic medications can be a lifesaver in many cases yet, it is well-documented that in almost half of patients these medications show limited effectiveness (Pouget et al., 2014; Alboni et al., 2016). This problem is often termed as “first failure.” The workers that experience first failure are burdened with a prolonged trial-and-error period to determine the correct medication and dose. Others can be burdened with adverse drug reactions (Adverse Drug Reaction, 2018). Genomic profiling that identifies the ineffectiveness of prescription medication could be an effective solution to reduce long trial and error process for selecting the right medication for the right patient (Singh, 2015).

We showed that all workers referred to us by the disability managers had one or more genetic mutations impacting their medication effectiveness (Hyman, 2000). We noted a strong correlation of our genetic mutation score with medication effectiveness scores indexing poor mood toward work tasks and reported side effects (Malhotra and Murphy, 2004). We can infer that their genomic profile had a deleterious impact on their treatment outcomes and their ability to function at work. We found that prescription changes based on pharmacogenomic profile resulted in profound four-fold improvement in treatment effectiveness score in our patient group (Bradley et al., 2018).

There have been suggestions to incorporate pharmacogenomic profiling in patients on mental health-related disability (Biogeni, 2019). Pharmacogenomics has been on radar of disability management professionals for some time as they recognize that personalized medicine concepts are yet to be adopted comprehensively in clinical management of mental health conditions (Stuckey, 2015). Nevertheless, pharmacogenomics incorporation in disability management is expected to have high returns in terms of reducing time to return-to-work and disability management costs (Schwartz, 2009).

This study also sheds light on importance of summarizing evidence and developing tools for pharmacogenomic profiling. For instance, the 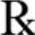 Report^®^ test identifies variations in multiple genes involved in the drug's efficacy that have been published or available in gray literature from the Food and Drug Administration (FDA), the Pharmacogenomics Knowledgebase (PharmGKB), the Clinical Pharmacogenetics Implementation Consortium (CPIC) and the Dutch Pharmacogenetics Working Group (DPWG). Unlike many other tests, the 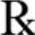 Report^®^ includes genetic variations related to both pharmacokinetics and pharmacodynamics (Fabbri, 2017; Stahl, 2017). In addition, prescription changes following our report is an indirect indicator that disability managers and physicians might have found our 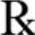 Report^®^ as “easy to read and interpret.”

Our study has some limitations. Firstly, the patients that were referred to us were already being suspected to have genomic profile affecting their treatment effectiveness. So, our study could over-estimate the potential benefits of pharmacogenomics. Secondly, a large proportion of patients were lost to follow-up. Thirdly, our study is an observational study. For assessing efficacy of pharmacogenomic profiling, we plan to conduct a randomized controlled trial in near future. The smaller sample size prevented us from including individual prescription changes because of risk of re-identification. In future, we expect to have larger sample size to indicate common prescription changes prompted by such testing in these patients. Lastly, we were not provided access to time to return-to-work indicators that could have helped us to assess the economic benefits of pharmacogenomic profiling further.

In conclusion, we suggest that agencies handling mental health disability claims should consider pharmacogenomic profiling in patients under their management (Schwartz, 2009). Such strategies could have a significant impact on drug adherence (Fagerness et al., 2014), quality of life (Hornberger et al., 2015), and cost savings (Brown et al., 2017) in their patients. The 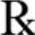 Report^®^ can be one of the tools to help disability managers positively impact treatment outcomes in workers on mental health-related disability.

## Data Availability Statement

The original contributions presented in the study are included in the article, further inquiries can be directed to the corresponding author.

## Ethics Statement

The protocol was reviewed by the Research Ethics Board of the Sunnybrook Health Sciences Centre (Toronto, ON) with reference # PIN 3336 (Approved Nov 30, 2020). The patients/participants provided their written informed consent to participate in this study.

## Author Contributions

SA: conception or design of the work and data collection. RT, MF, and UA: data analysis and interpretation. SA, MF, and UA: drafting the article. MF and UA: critical revision of the article. SA, RT, UA, and MF: final approval of the version to be published. All authors had full access to all of the data in the study and take responsibility for the integrity of the data and the accuracy of the data analysis.

## Conflict of Interest

SA and MF were employed by company Personalized Prescribing Inc., Canada. RT was employed by company Apotex Pharmaceuticals, Canada. The remaining author declares that the research was conducted in the absence of any commercial or financial relationships that could be construed as a potential conflict of interest.

## Publisher's Note

All claims expressed in this article are solely those of the authors and do not necessarily represent those of their affiliated organizations, or those of the publisher, the editors and the reviewers. Any product that may be evaluated in this article, or claim that may be made by its manufacturer, is not guaranteed or endorsed by the publisher.
